# Effects of ZnT8 on epithelial-to-mesenchymal transition and tubulointerstitial fibrosis in diabetic kidney disease

**DOI:** 10.1038/s41419-020-2731-6

**Published:** 2020-07-17

**Authors:** Xiuli Zhang, Tingwen Guan, Boxuan Yang, Harvest F. Gu, Zhihong Chi

**Affiliations:** 1 Department of Nephrology, Second People’s Hospital, The First Affiliated Hospital of Shenzhen University, 518000 Shenzhen, Guangdong Province P.R. China; 2 Department of Pathophysiology, China Medical University, 110001 Shenyang, Liaoning Province P.R. China; 3 Center for Pathophysiology, School of Basic Medicine and Clinical Pharmacy, China Pharmaceutical University, 210009 Nanjing, Jiangsu Province P.R. China

**Keywords:** Biological sciences, Cell biology

## Abstract

Zinc transporter 8 (ZnT8) transports zinc ions for crystallization and storage of insulin in pancreatic beta-cells and ZnT8 dysfunction is involved in pathogenesis of diabetes. The current study aimed to investigate whether ZnT8 has effects in pathophysiology of diabetic kidney disease (DKD) by using animal models for diabetes, including STZ-induced diabetic, db/db, ZnT8-KO, ZnT8-KO-STZ and ZnT8-KO-db/db mice. Results demonstrated that urine albumin to creatinine ratio and epithelial-to-mesenchymal transition (EMT) were increased in kidneys of ZnT8-KO-STZ and ZnT8-KO-db/db mice compared with C57BL/6 J and ZnT8-KO mice, while serum TGF-β1, IL-6, and TNF-α levels were elevated in parallel. In kidneys of mice intercrossed between ZnT8-KO and STZ-induced diabetic or db/db mice, these three inflammatory factors, ACR and EMT were also found to be increased compared with C57BL/6J, db/db and ZnT8-KO mice. Furthermore, ZnT8 up-regulation by hZnT8-EGFP reduced the levels of high glucose (HG)-induced EMT and inflammatory factors in normal rat kidney tubular epithelial cell (NRK-52E cells). Expression of phosphorylated Smad2/Smad3 was up-regulated after HG stimulation and further enhanced by ZnT8 siRNA but down-regulated after hZnT8-EGFP gene transfection. The current study thus provides the first evidence that ZnT8 protects against EMT-tubulointerstitial fibrosis though the restrain of TGF-β1/Smads signaling activation in DKD.

## Introduction

Diabetic kidney disease (DKD, previously termed as diabetic nephropathy) is the most common cause of chronic kidney failure and end stage kidney disease^1,2^. Clinical manifestation of DKD is characterized by hyperfiltration, renal hypertrophy, progressively increasing proteinuria, and deterioration of renal function^3^. Loss of podocytes, enhanced glomerular and tubulointerstitial accumulation of extracellular matrix (ECM), apoptosis of renal tubular epithelial cells, infiltration of blood mononuclear cells, and proliferation of interstitial mesenchymal cells are the principal pathological features of DKD, which has been detected in renal biopsy samples from patients with DKD^4^. Tubulointerstitial fibrosis is the final common pathway of the majority of chronic progressive renal diseases, including DKD, which involves expansion of interstitial fibroblasts, myofibroblast activation and ECM accumulation, leading to the loss of normal kidney function and, ultimately, renal failure^5^. EMT of mature tubular epithelial cells appears as the central point in the early pathogenesis of the development and progression of renal interstitial fibrosis in DKD. The character is that epithelial cells lose their epithelial specific markers, undergo cytoskeletal remodeling, and gain a mesenchymal phenotype^6^. Furthermore, accumulating evidence has demonstrated that inflammatory molecules and mediators are important in the pathogenesis of DKD. Several inflammatory cytokines such as tumor necrosis factor-alpha (TNF-α), interleukin-6 (IL-6) and transforming growth factor beta 1 (TGF-β1) are found to be associated with glomerular and tubulointerstitial damage in the progresses of DKD^7–12^.

Zinc transporter 8 (ZnT8 also known as SLC30A8) is localized in insulin secretory granules in the islets of Langerhans and facilitates the accumulation of zinc from the cytoplasm into intracellular vesicles in pancreatic beta-cells^13,14^. Recently, we have reported that ZnT8 also transports zinc into Leydig cell mitochondria with gonadotropin stimulation and plays a role in testosterone production via the PKA signaling pathway^15^. ZnT8 has genetic, epigenetic and biological effects in the pathogenesis of diabetes^16^. ZnT8 auto-antibodies (ZnT8A) are considered as the most recently discovered and least-characterized islet auto-antibodies to predict risk of future type 1 diabetes (T1DM)^17–19^. Genetic association studies, including ours have demonstrated that the common single nucleotide polymorphisms in the SLC30A8 gene confer the risk susceptibility to type 2 diabetes (T2DM), while the rare loss-of-function variants in the gene have protective effects in the disease^20,21^. An epigenetic study from our group has reported that increased DNA methylation of the SLC30A8 gene promoter is associated with T2DM^22^. However, the question whether ZnT8 has effects in diabetic complications, including DKD has been proposed. In 2012, DeNiro et al. have suggested that ischemic retinopathy may be mediated by aberrant Zn^2+^ homeostasis caused by ZnT8 downregulation^23^. Later on, Pinna et al. have showed that T1DM patients have higher rates of positive antibodies against MAP/ZnT8 peptides, but failed to find the correlation between the presence of these antibodies and the severity degree of high-risk proliferative diabetic retinopathy^24^. Xu et al. have demonstrated that erythropoietin (EPO) maintains zinc homeostasis through activating the ERK pathway and downregulating HIF-1α (hypoxia inducible factor-1α), and thus upregulating ZnT8 expression in diabetic retinas^25^. Based upon what described above, we have a hypothesis that ZnT8 may play an important role in the pathogenesis of DKD.

In the current study, we initially designed and performed in vivo experiments with non-diabetic control, ZnT8-KO, STZ-induced (animal model for T1DM) and ZnT8-KO-STZ mice and then with db/db (animal model for T2DM) and ZnT8-KO-db/db mice. We further carried out in vitro experiments with high glucose (HG)-stimulated EMT in normal rat kidney tubular epithelial cell (NRK-52E cells) by using transfection and RNA interference (RNAi) protocols. The aims were to explore the effects of ZnT8 on EMT-tubulointerstitial fibrosis in DKD and to understand its underlying molecular mechanism.

## Results

### Metabolic and biochemical parameters in the studied mice

STZ-induced diabetic mouse is an animal model for T1DM. In the initial experiments, ZnT8-KO, STZ-induced and ZnT8-KO-STZ diabetic mice were studied. As seen in Table 1A, compared with non-diabetic control (C57BL/6J) and ZnT8-KO mice, STZ-induced diabetic and ZnT8-KO-STZ mice were found to be exhibited diabetic symptoms such as increase in diet, drink and urine while body weight loss. Fasting blood glucose (FBG) levels in STZ-induced and ZnT8-KO-STZ diabetic mice were significantly higher than controls. After injection of STZ for 16 weeks, blood serum creatinine (Cr), urea nitrogen (BUN) and urine albumin-to-creatinine ratio (UACR) were increased in STZ-induced and ZnT8-KO-STZ diabetic mice, while ZnT8-KO-STZ mice exhibited the highest Cr, BUN and UACR levels among the groups.

**Table 1:** (A) Metabolic and biochemical parameters in Control, ZnT8-KO, STZ-induced and ZnT8-KO-STZ diabetic mice; (B) Metabolic and biochemical parameters in Control, ZnT8-KO, db/db and ZnT8-KO-db/db mice.

A	Cont	ZnT8-KO	STZ-induced	ZnT8-KO-STZ
FBG (mM)	5.21 ± 0.23	5.98 ± 0.17	23.48 ± 4.29**	26.75 ± 5.11**
Body weight (g)	26.3 ± 1.22	27.2 ± 2.31	21.06 ± 2.67*	22.32 ± 2.39*
Kidney weight (g)	0.32 ± 0.07	0.34 ± 0.26	0.33 ± 0.05	0.33 ± 0.06
BUN (mM)	6.23 ± 0.62	6.98 ± 0.43	23.37 ± 5.04**	25.09 ± 7.23**
Cr (µM)	27.12 ± 3.79	28.30 ± 3.25	79.36 ± 7.98**	80.03 ± 8.46**
UACR (µg/mg)	19.9 ± 2.03	21.32 ± 2.39	223.90 ± 10.78**	269.70 ± 11.36**^,#^
TGF-β1 (pg/ml)	1.94 ± 0.43	1.89 ± 0.20	4.46 ± 0.75**	8.85 ± 0.95**^,##^
IL-6 (pg/ml)	2.63 ± 0.41	2.82 ± 0.44	5.77 ± 0.88**	6.84 ± 0.96**^,#^
TNF-α (pg/ml)	9.37 ± 1.01	9.58 ± 0.65	29.71 ± 1.67**	42.97 ± 2.15**^,##^

B	Cont	ZnT8-KO	db/db	ZnT8-KO-db/db
FBG (mM)	5.19 ± 0.18	5.71 ± 0.12	39.70 ± 2.62**	43.90 ± 4.21**^,†^
Body weight (g)	25.7 ± 1.09	26.5 ± 2.03	43.10 ± 2.33*	44.27 ± 3.06*
Kidney weight (g)	0.32 ± 0.09	0.33 ± 0.13	0.34 ± 0.09	0.34 ± 0.11
BUN (mM)	5.31 ± 0.19	5.62 ± 0.22	10.79 ± 0.19**	13.01 ± 8.32**^,†^
Cr (µM)	12.78 ± 1.98	13.15 ± 1.94	68.46 ± 9.17**	92.38 ± 10.35**^,†^
UACR (µg/mg)	9.91 ± 0.92	11.32 ± 1.39	308.63 ± 1 2.29**	357.46 ± 13.17**^,#^
TGF-β1 (pg/ml)	1.97 ± 0.12	1.91 ± 0.17	5.03 ± 0.83**	7.02 ± 0.69**^,‡^
IL-6 (pg/ml)	2.91 ± 0.36	3.19 ± 0.35	7.39 ± 0.36**	6.87 ± 0.72**^,‡^
TNF-α (pg/ml)	10.21 ± 1.02	9.34 ± 0.83	29.27 ± 2.29**	39.77 ± 1.76**^,‡^

Data are presented as means ± SEM.

*P < 0.05 and **P < 0.01 vs. Control group; ^#^*P* < 0.05 and ^##^*P* < 0.01 vs. STZ-induced diabetic group. Mice in each group, *n* = 8.

*P < 0.05 and **P < 0.01 vs. Control group; ^†^*P* < 0.05 and ^‡^*P* < 0.01 vs. db/db group, Mice in each group, *n* = 6.

Db/db mouse is another animal model and for T2DM. The next experiments with Control, ZnT8-KO, db/db and ZnT8-KO-db/db mice were done. In Table 1B, db/db and ZnT8-KO-db/db mice exhibited significantly increased levels of not only FBG but also body weight compared with control and ZnT8-KO mice, while db/db and ZnT8-KO-db/db mice demonstrated higher BUN, Cr and UACR than what in control and ZnT8-KO mice. In both initial and next experiments, the comparison analyses between control and ZnT8-KO mice were performed and no significant difference between control and ZnT8-KO mice was seen.

### Serum levels of TGF-β1, IL-6 and TNF-α in the studied mice

Serum levels of TGF-β1, IL-6 and TNF-α in all studied mice were analyzed with Elisa protocols in initial and next experiments, respectively, and data were included in Table 1A, B. Serum levels of TGF-β1, IL-6 and TNF-α in STZ-induced diabetic, db/db, ZnT8-KO-STZ and ZnT8-KO-db/db mice were significantly elevated compared with control or ZnT8-KO mice, while these three inflammatory factors had similar levels in ZnT8-KO-STZ and ZnT8-KO-db/db mice.

### Effects of ZnT8 in EMT-tubulointerstitial fibrosis on kidneys of the studied mice

**Fig. 1:**
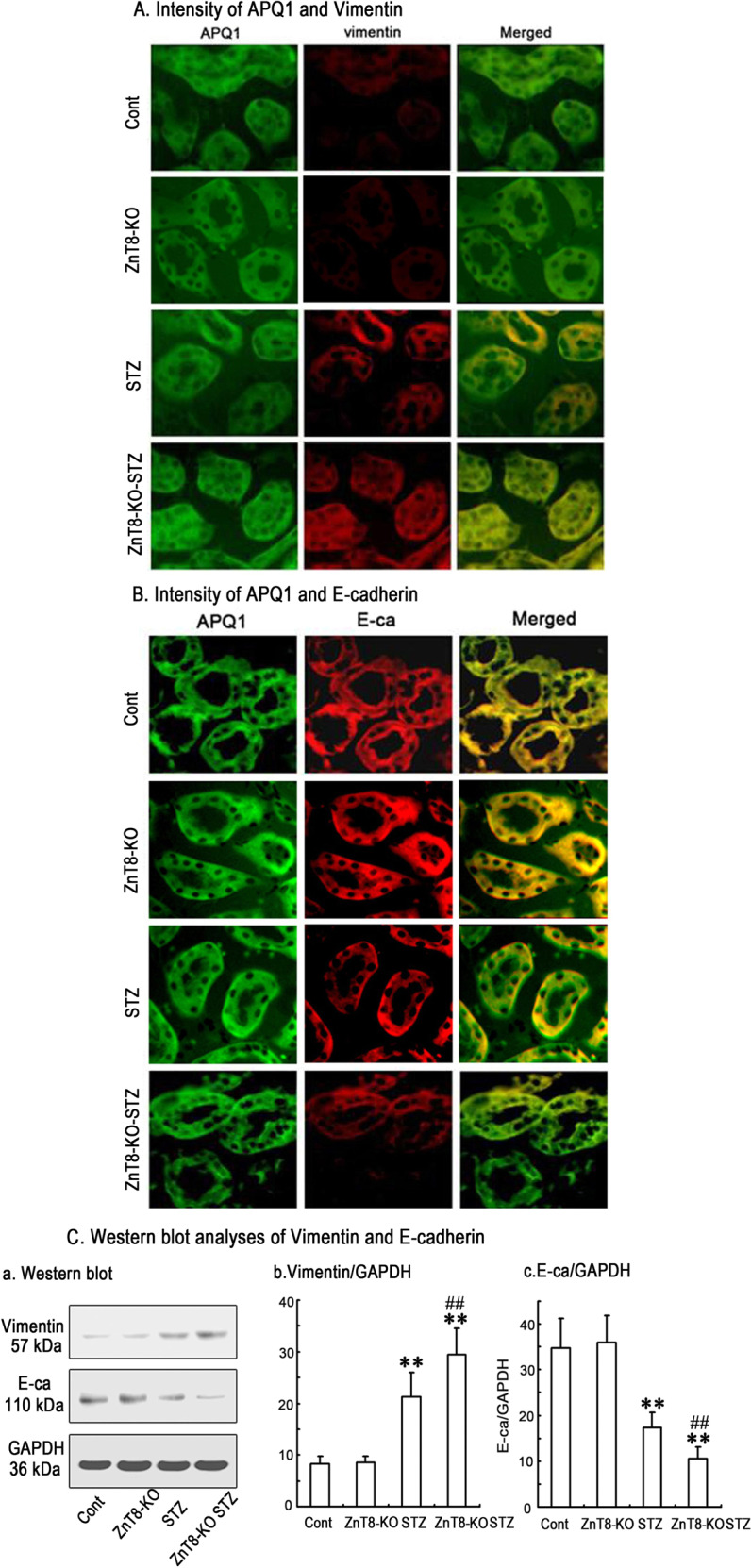
Vimentin and E-cadherin expression levels in proximal tubular cells of kidneys in Control, ZnT8-KO, STZ-induced diabetic and ZnT8-KO-STZ mice. In the initial experiments, immunofluorescence staining images demonstrated the localization of Vimentin (red) and E-cadherin (red) in the proximal tubular cells of kidneys of Control (a1-3), ZnT8-KO mice (b1-3), STZ-induced diabetic (c1-3) and ZnT8-KO-STZ mice (d1-3) and data are represented in **a**, **b**, respectively. AQP1 (green) is the marker for tubular cells. Original magnification, ×400. Data from Western blotting analyses were followed in **C**. Relative expression of Vimentin and E-cadherin was calculated and normalized to the loading controls. The experiments were repeated for three times (**P < 0.001 vs. Control mice and ^##^*P* < 0.001 vs. STZ-induced diabetic mice).

To investigate the effect of ZnT8 in EMT-tubulointerstitial fibrosis in kidneys, expression and localization of anti-aquaporin-1 (APQ1, a proximal tubule marker) and Vimentin (a mesenchymal marker) and E-cadherin (E-ca, an epithelial specific marker) in renal interstitium of control, STZ-induced and ZnT8-KO-STZ diabetic mice were detected by using the double-immunofluorescence staining in the initial experiments. Figure 1a implicated that Vimentin protein (red) was virtually absent in kidneys of control mice or ZnT8-KO mice (Fig. 1a, a1–3, b1–3) compared with STZ-induced and ZnT8-KO-STZ diabetic mice. Furthermore, the intensity of Vimentin staining in interstitial compartment of ZnT8-KO-STZ diabetic mice (Fig. 1a, d1–3) was stronger than what in STZ-induced diabetic mice (Fig. 1a, c1–3). Unlike expression of Vimentin, expression of E-cadherin of the tubular epithelial cells in kidneys of STZ-induced and ZnT8-KO-STZ diabetic mice was decreased compared with control mice or ZnT8-KO mice (Fig. 1b, a1–d3). Consistently, analyses of kidney tissues by Western blot showed that expression levels of Vimentin were significantly increased but E-cadherin decreased in kidneys of ZnT8-KO-STZ mice compared to control or ZnT8-KO mice (Fig. 1c).

**Fig. 2:**
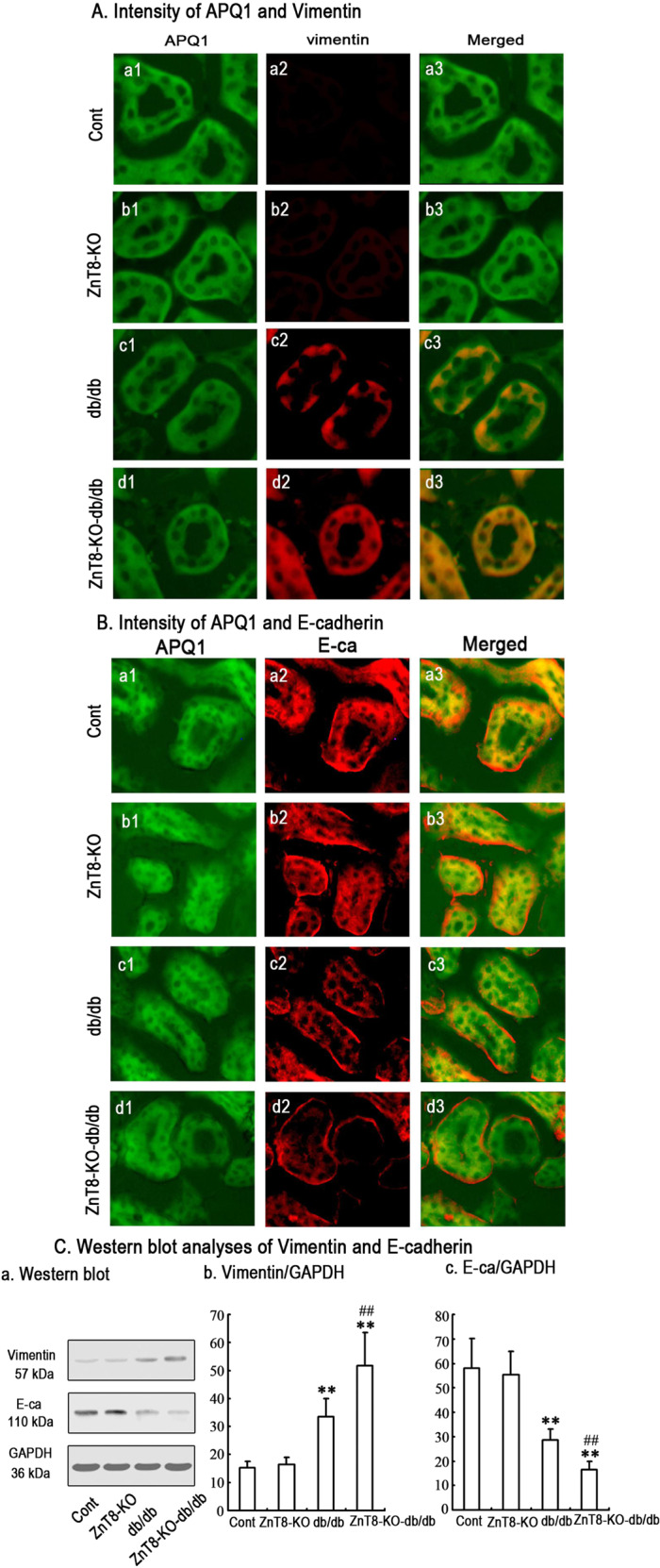
Vimentin and E-cadherin expression levels in proximal tubular cells of kidneys in Control, ZnT8-KO, db/db and ZnT8-KO-db/db mice. In the next experiments, immunofluorescence staining implicated the localization of Vimentin (red) and E-cadherin (red) in the proximal tubular cells of kidneys of Control (a1-3), ZnT8-KO mice (b1-3), db/db (c1-3) and ZnT8-KO-db/db mice (d1-3) and data are represented in **a**, **b**, respectively. AQP1 (green) is the marker for tubular cells. Original magnification, ×400. Data from Western blotting analyses in **c** were attached as below. Relative expression of Vimentin and E-cadherin was calculated and normalized to the loading controls. The experiments were repeated for three times. The experiments were repeated for three times (**P < 0.001 vs. Control mice; ^##^*P* < 0.001 vs. db/db mice).

In next experiments, the similar analyses were further conducted in control, db/db and ZnT8-KO-db/db mice and the results were represented in Fig. 2a–c. Similarly, Vimentin expression was found to be increased while E-cadherin expression decreased in kidneys from db/db and ZnT8-KO-db/db mice.

### Effects of ZnT8 on HG-induced EMT-tubulointerstitial fibrosis in NRK-52E cells

To understand the mechanism underlying the effect of ZnT8 in EMT-tubulointerstitial fibrosis in kidneys of the studied mice, the experiments for HG-stimulated on EMT by modulating ZnT8 expression in NRK-52E cells were further performed with transfection protocol combined with RNAi techniques. The highest expression levels of ZnT8 after transfection of hZnT8-EGFP was found at 72 h (Supplemental Fig. 1). We confirmed that ZnT8 protein expression was reduced to ~13% of the normal level by using Western blotting analyses after ZnT8siRNA transfection (Supplementary Fig. 1).

**Fig. 3:**
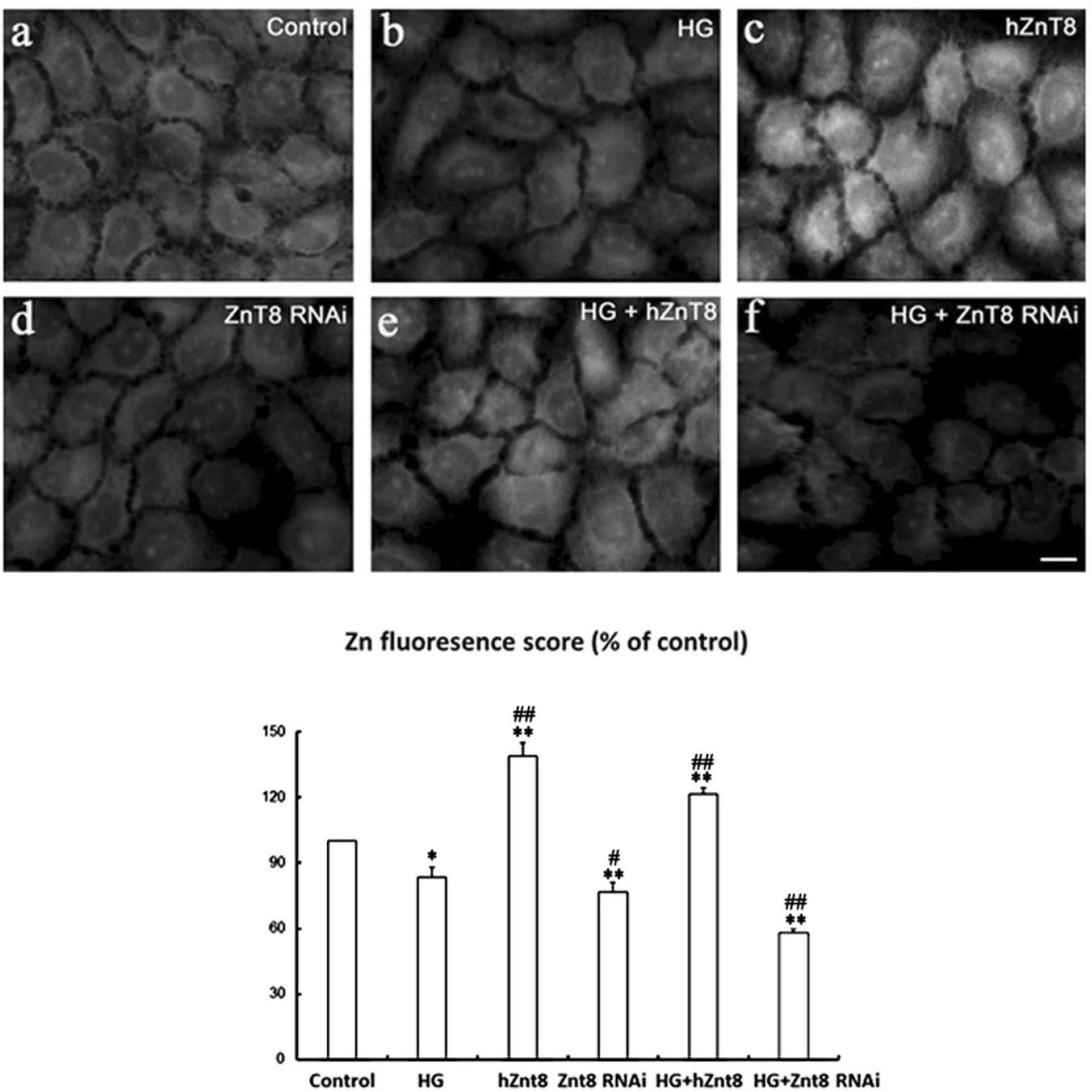
Free zinc distribution in NRK-52E cells. NRK-52E cells were treated, respectively, with scrambled control, HG, hZnT8, ZnT8 RNAi, HG+hZnT8 and ZnT8 RNAi. Free zinc distribution in the cells was then analyzed with TSQ assays (**a**–**f**). Relative zinc fluorescing scores each group were summarized as below (*P < 0.05 and **P < 0.001 vs. control group; ^#^*P* < 0.05 and ^##^*P* < 0.001 vs. HG).

After transfection of hZnT-8-pcDNA3.1/myc-HisA or ZnT8-siRNA, the NRK-52E cells were then treated with HG for 48 h. To evaluate the effects of ZnT8 on Zinc distribution, we performed TSQ fluorescence staining in the cells. A strong granular zinc staining was found in the perinuclear region of hZnT8 and HG/hZnT8 groups (Fig. 3). The highest score of zinc fluorescence staining was detected in hZnT8 group (Fig. 3c). The fluorescent intensity in HG/ZnT8 RNAi group was weakest (Fig. 3f).

**Fig. 4:**
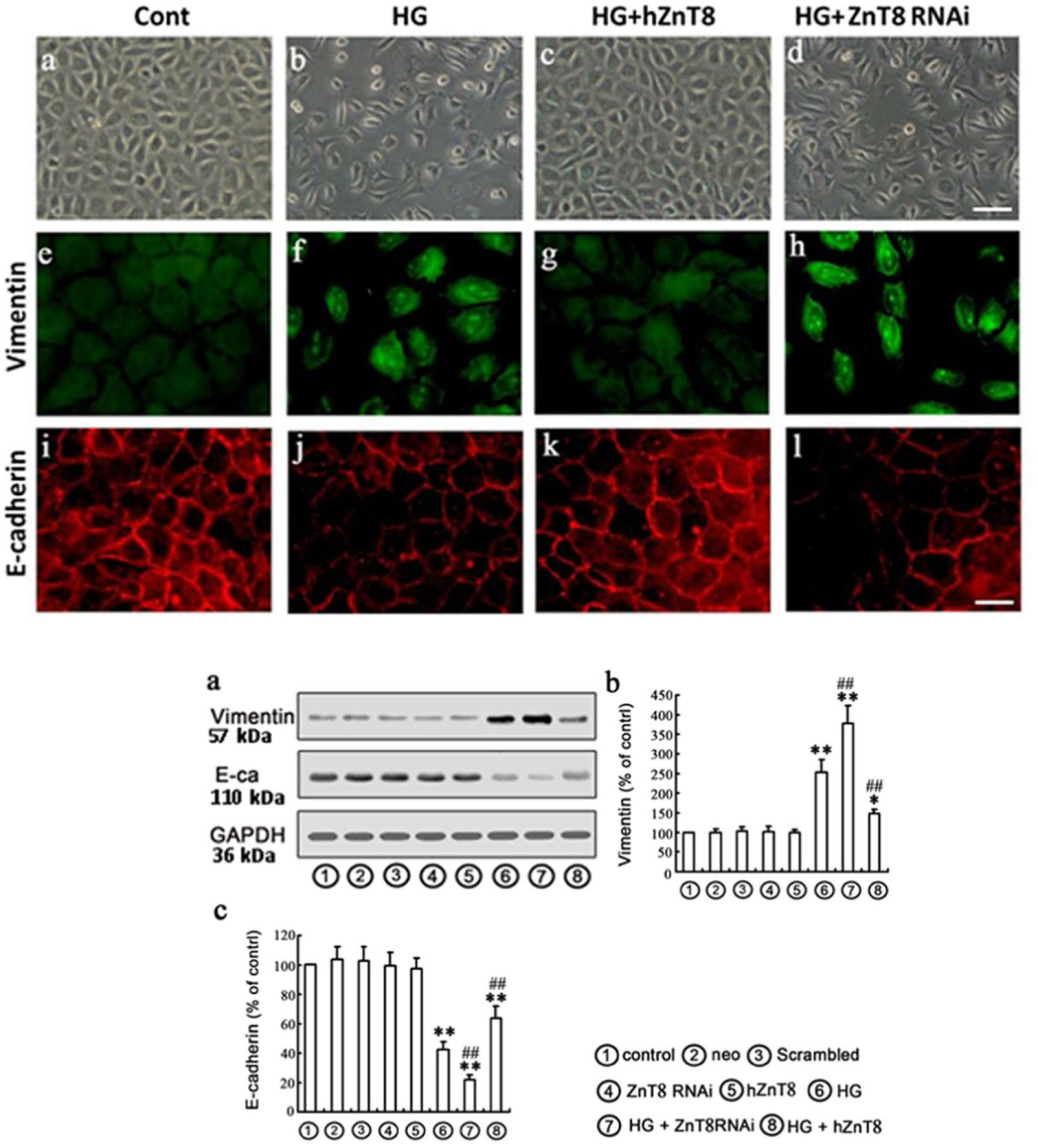
Effects of ZnT8 on EMT markers Vimentin and E-cadherin in NRK-52E cells. Protein expression EMT markers, including Vimentin (green) and E-cadherin (red), in NRK-52E cells were detected with immunofluorescence chemistry (above, magnification was ×400) and Western blot (below), and represented under the conditions of Control, Neo (empty vector), Scrambled, ZnT8 RNAi, hZnT8, HG, HG plus hZnT8 or ZnT8 RNAi in **a**–**c** (below), respectively, (*P < 0.05 and **P < 0.001 vs. Control group, ^##^*P* < 0.001 vs. HG).

HG treatment in NRK-52E cells for 48 h led to cell morphological changes to a fibroblast-like shape (Fig. 4b–d above). Meanwhile, expression of Vimentin was up-regulated (Fig. 4f–h above), while E-cadherin was down-regulated (Fig. 4j–l above), whereas this HG-induced EMT can be attenuated by over-expressing ZnT8 in NRK-52E cells. The protein expression of Vimentin and E-cadherin analysis by Western blot represented under the conditions of control, neo, scrambled, ZnT8 RNAi, hZnT8, HG, HG plus hZnT8 or ZnT8 RNAi, respectively. The results suggested that ZnT8 expression is associated with HG-induced EMT in a way that over-expressing ZnT8 in NRK-52E cells significantly decreased HG-induced EMT, as evidenced by the reduced up-regulation of Vimentin and ameliorated expression of E-cadherin (Fig. 4a–c below).

### Effects of ZnT8 on HG-induced TGF-β1/Smad pathway

**Fig. 5:**
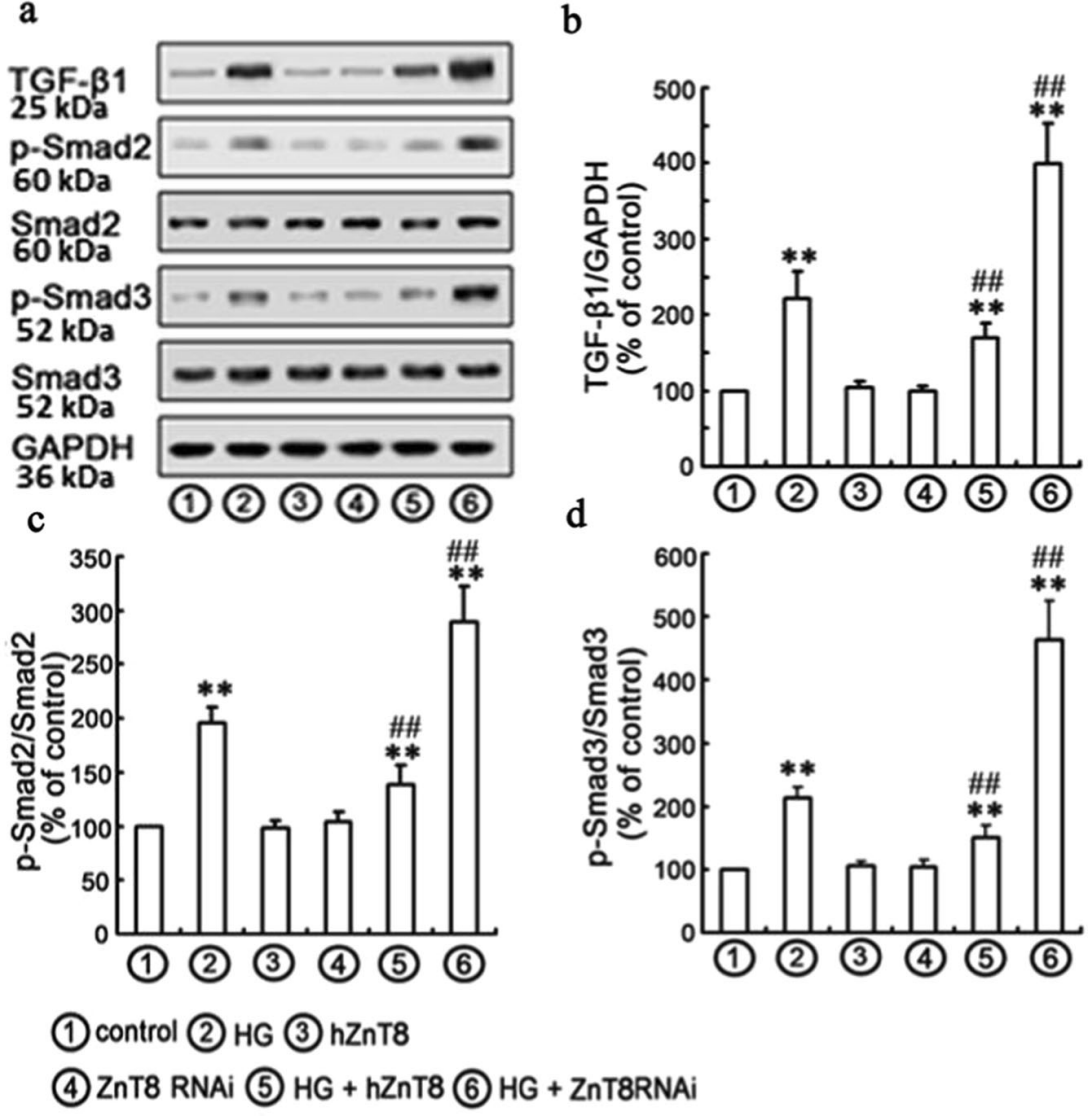
Effects of ZnT8 on HG-induced TGF-β1/Smad signaling pathway in NRK-52E cells. Western blotting analyses were performed with the antibodies indicated **a**. Under the conditions of Control, HG, hZnT8, ZnT8 RNAi, HG plus hZnT8 and ZnT8 RNAi, relative expression levels of TGF-β1, p-Smad2, and p-Smad3 calculated and normalized to the loading control and represented in **b**–**d** (**P < 0.001 vs. Control; ^#^*P* < 0.05 and ^##^*P* < 0.001 vs. HG).

**Fig. 6:**
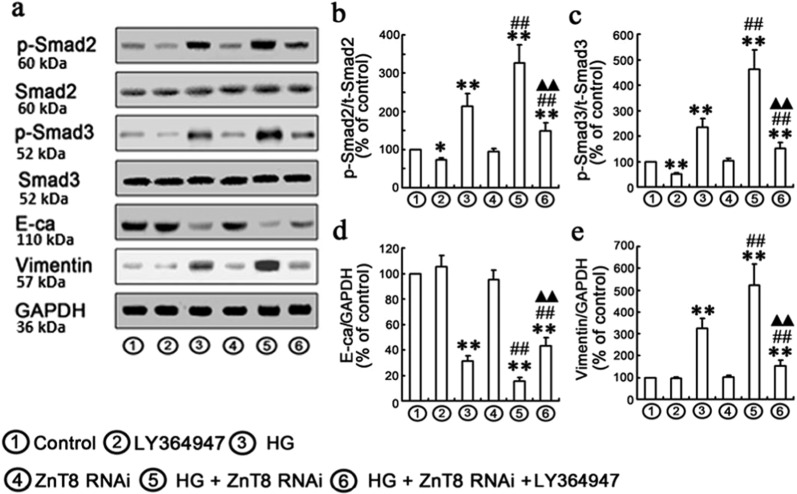
Effects of ZnT8 on Smad2/3 and p-Smad2/3 activities and EMT markers in NRK-52E cells. NRK-52E cells were incubated with or without 10 μM LY364947 for 1 h and subsequently exposed to ZnT8 RNAi for 24 h and HG for 48 h. Protein expressions of EMT markers (Vimentin and E-cadherin), Smad2/3 and p-Smad2/3 were analyzed with Western blot (**P < 0.01 vs. Control; ^#^*P* < 0.05 and ^##^*P* < 0.01 vs. HG; ^▲▲^*P* < 0.01 vs. HG/ZnT8 RNAi).

TGF-β1/Smad pathway plays an important role in the pathogenesis of ECM accumulation in DKD^26^. In the current study, the protein levels of TGF-β1, p-Smad2 and p-Smad3 were found to be significantly increased in HG-exposed NRK-52E cells (Fig. 5a, d). Therefore, transfected overexpressed or knockdown ZnT8 gene was applied to determine if ZnT8-mediated protection against HG-induced EMT by inhibition of TGF-β1/Smad pathway. After transfection of ZnT8 siRNA or hZnT-8-pcDNA3.1/myc-HisA, NRK-52E cells were treated with HG for 48 h. The levels of TGF-β1, p-Smad2 and p-Smad3 were found to be significantly increased in HG/ZnT8 RNAi group compared with Control or HG group. Conversely, over-expression of ZnT8 significantly decreased HG-induced levels of TGF-β1, p-Smad2 and p-Smad3 (Fig. 5a, d). To further confirm the role of TGF-β1/Smad signaling in the protective effects of ZnT8 on HG-induced EMT in NRK-52E cells, 10 μM LY364947 (the inhibitor of TGF-βRI) was added to the medium of NRK-52E cells for 1 h^27^. Data indicated that co-treatment of LY364947 effectively inhibited the levels of p-Smad2, p-Smad3 and HG-induced EMT (Fig. 6a–e).

## Discussion

By using ZnT8-KO, STZ-induced, db/db, ZnT8-KO-STZ and ZnT8-KO-db/db mice and NRK-52E cells, we have performed in vivo and in vitro experiments to investigate the effects of ZnT8 on EMT-tubulointerstitial fibrosis. The findings mainly include: first, expression level of EMT marker Vimentin was increased and E-cadherin decreased in kidneys of ZnT8-KO-STZ and ZnT8-KO-db/db mice compared with diabetic STZ and db/db mice. Furthermore, the circulating inflammation factors, including TGF-β1, IL-6 and TNF-α, in serum samples and ACR in urine samples were also increased. In parallel, expression of EMT markers in NRK-52E under HG was found to be up-regulated. Overexpression of ZnT8 gene by hZnT8-EGFP reduced the HG-induced EMT and inflammatory factors. Second, expression of phosphorylated Smad2 and Smad3 was up-regulated after HG stimulation and further enhanced by ZnT8 siRNA but down-regulated after hZnT8-EGFP gene transfection.

ZnT8 is known to catalyze the extrusion of zinc from the cell cytosol into the extracellular space or intracellular organelles^28^. It is well known that ZnT8 is expressed in pancreatic islets and important for diabetes research^13,14,29^. This gene, however, is also expressed in other tissues, while kidney is the second rich expressed organ (please see https://www.genecards.org/Search/Keyword?queryString=Znt8). Furthermore, we have recently found that ZnT8 is expressed in Leydig cells, transports zinc into Leydig cell mitochondria with gonadotropin stimulation^15^. A global loss of ZnT8 gene in mice rather than β-cell-specific knockdown of ZnT8, leads to glucose intolerance, insulin resistance and worsen obesity in those high-fat diet feeding^30^. Whereas transgenic mice with overexpressing ZnT8 gene selectively in pancreatic β-cells showed a significant improvement in glucose tolerance compared to control animals^27^. STZ-induced diabetic mice exhibit typical symptoms for T1DM, while db/db mouse is an animal model for T2DM. In the current study, we have studied with both animal models. Importantly, we have generated ZnT8-KO-STZ and ZnT8-KO-db/db mice, which exhibit significant increase in fasting blood glucose and albuminuria compared to diabetic animals. Furthermore, double-immunofluorescence staining and Western blot analyses indicate that EMT-tubulointerstitial fibrosis is higher in ZnT8-KO-STZ and ZnT8-KO-db/db mice than what in STZ and db/db mice. Therefore, data from the current study may suggest that ZnT8 have the similar effects on EMT-tubulointerstitial fibrosis in animal models for both T1DM and T2DM. It is indeed important to investigate the location of expressed ZnT8 in kidney tissues. The glomerulus, particularly the mesangium, has been the focus of intense investigation in diabetes and DKD. Tubulointerstitial injury, however, is also a major feature of DKD and an important predictor of renal dysfunction. Continued exploration into tubulointerstitial disease in addition to glomerular injury in diabetes may help provide further insights into the pathogenesis of DKD and additional targets for therapeutic intervention^31^. In the present study, we carried out the experiments and data implicated that ZnT8 was mainly expressed in renal epithelial tubular cells but very weakly in glomerulus or podocyte (please see Supplementary Fig. 2). However, the resolution of the images was not high due to the limitation of immunofluorescence staining technique. Therefore, it is necessary to ascertain the location of ZnT8 in kidneys with higher resolution technique such as the micro-dissection.

EMT plays an important role in tubulointerstitial fibrosis, which is a hallmark of DKD and identification of the mechanisms of EMT activation and targeted to reverse will be informative for understanding the pathogenesis of DKD^32^. One hand, acute cytokine exposure of pancreatic β-cells reduced ZnT8 expression and intracellular labile zinc, impairing β-cell function^33^. On another hand, there is a close relationship between down-regulation of zinc transporter expression, zinc homeostasis and inflammatory response, which may contribute to the development of diabetes and DKD^16,21^. A previous study has demonstrated that inflammation or TNF-α-induced protein-3 dysfunction may be involved in the pathogenesis of diabetes via ZnT8 expression, besides from pancreatic islet cell apoptosis, in which protects ZnT8 against pro-inflammatory cytokine-induced downregulation^34^. Evidence that TGF-β1 plays significant role in EMT of renal epithelial cells, so we as to focus on the TGF-β1 in effect of ZnT8 on EMT of renal epithelial cells. In the current study, we demonstrated that inflammation factors, including IL-6, TNF and TGF-β1 in serum samples of ZnT8-KO-STZ and ZnT8-KO-db/db mice were significantly increased, which suggested that these inflammation factors together with ZnT8 dysfunction may play an important role in the progression of DKD. ZnT8 overexpression with hZnT-8-pcDNA3.1/myc-hisA transfection and siRNAs targeting ZnT-8 were used in NRK52-E cells. First, we found knockdown of ZnT8 by siRNAs significantly increased the level of TGF-β1. Conversely, overexpression of ZnT-8 decreased the TGF-β1 level. In addition, results also suggest that ZnT8 expression is associated with HG-induced EMT in a way that over-expressing ZnT8 in NRK-52E cells significantly decreased HG-induced EMT, as evidenced by the reduced up-regulation of vimentin and ameliorated expression of E-cadherin. Furthermore, we also found that the intracellular zinc accumulation was decreased after knockdown of ZnT-8 while overexpression of ZnT8 increased in intracellular zinc accumulation which followed by reversed HG-induced EMT. These data may provide further evidence of a role for the intracellular Zn^2+^ contributing to Zn^2+^ inhibit the TGF-β1 and EMT though ZnT8 mediated.

TGF-β1 plays significant role in EMT of renal epithelial cells and the activation its downstream TGF-β1/Smad signaling activation is required in TGF-β1-induced EMT in human proximal tubular epithelial cells^32,35^. When TGF-β binds to its receptor TGF-βR1, Smad2/3 is phosphorylated and binds with Smad4, followed by translocation into the nucleus, where these complexes activate the transcription of pro-fibrotic genes^26,35^. Smad4, a TGF-β signal mediator, is required for the occurrence and maintenance of EMT and in renal fibrosis, inactivation or decreased expression of Smad4 is frequently observed^36^. Previous study showed that N6-(2-Hydroxyethyl) adenosine decreased lipopolysaccharide-induced inflammatory cytokine level in rat renal fibroblast cells and TGF-β1-induced fibroblast activation in NRK-49F cells by modulating NF-ΚB and TGF-β1/Smad signaling^37^. Other study demonstrated that Tisp40 plays a critical role in the TGF-β/ Smads pathway involved in this process. Hence, Tisp40 could be a useful therapeutic target in the fight against renal tubulointerstitial fibrosis^38^. Recently, we have reported that ZnT7 has a protective effect over EMT of tubular epithelial cells under HG condition and suggests that the inhibition of HG-induced EMT may be achieved through TGF-β/Smad pathway^32^. Because the TGF-β signaling pathway plays a central role in tubulointerstitial fibrosis, so we next investigated whether ZnT8 affected TGF-β pathway activity. To further explore the role of ZnT8 in EMT of tubular epithelial cells, in the current study, we have focused on the classic pro-fibrotic TGF-β signaling pathway. We found that knockdown of ZnT8 with siRNAs protocol significantly increased the luciferase activity of the Smad2 and 3. Conversely, overexpression of ZnT8 decreased the Smad2 and 3 activity. Inhibition of TGF-β1/Smad signaling activation by its specific inhibitor abrogated HG-induced activation of Smad2 and 3.

In conclusion, the current study for the first time demonstrates that ZnT8 has the protective effects against EMT-tubulointerstitial fibrosis though the restrain of TGF-β1/Smads signaling activation, which may provide a potential novel strategy for the prevention and treatment of EMT-tubulointerstitial fibrosis in DKD.

## Material and methods

### Animals and kidney tissue preparation

The animals used in the current study included control (C57BL/6J), ZnT8-KO, STZ-induced and ZnT8-KO-STZ mice in the first part of experiments and control, ZnT8-KO, db/db and ZnT8-KO-db/db mice in the second part of experiments. C57BL/6J male mice at 6 weeks were purchased from the Experimental Animal Center, China Medical University (Shenyang, China). Both C57BL/6N and C57BL/6J are derived from the same parental C57BL/6 strain, there are key genotypic and phenotypic differences between these two substrains. The C57BL/6J was the first and most extensively sequenced mouse genome. Therefore, large amounts of genetic and metabolic data have been generated using 6J^39,40^. The mice at 8 weeks of age were administered with STZ (150 mg/kg; Sigma-Aldrich; Merck Millipore, Darmstadt, Germany) diluted in 0.1 M citrate buffer (pH 4.5) as described previously^41^. The mice at 8 weeks serving as vehicle controls were given by intraperitoneal injection of the same volume of sodium citrate. Blood glucose levels for all mice were measured using an Accu-chek glucometer (Roche Diagnostics, Basel, Switzerland), while the mice with blood glucose >16 mmol/l were considered as diabetic one. At the time of sacrifice after injection STZ for 16 weeks, 24-h urines were collected in the metabolic cages. ZnT8-KO (C57BL/6 J) male mice at 12 weeks of age were obtained from GenOway, France. db/db (C57BL/KS.Cg-Lepr^db^) mice at 8 weeks of age were purchased from Model Animal Research Center of Nanjing University (MARC, Nanjing, China). ZnT8-KO male mice were subsequently intercrossed with female STZ-induced or db/db mice to generate ZnT8-KO-STZ and ZnT8-KO-db/db mice, respectively. All mice were housed in plastic cages with a controlled environment (22–25 °C; 50% humidity; and 12-h light/dark cycle) and had free access to food and distilled water. These animal models were generally considered to be used for study of diabetes and DKD^42^.

The mice used for experiments were terminally anesthetized with sodium pentobarbital (4%; Merck Millipore) and perfused with isotonic saline, followed by 4% paraformaldehyde in 0.1 M PBS (pH 7.4). The kidneys were removed and post-fixed with 4% paraformaldehyde overnight at 4 °C for use in immunofluorescent analysis and decapitated for Western blot analysis, respectively. The experimental procedures with mice were carried out by following the rules for experimental animals at China Medical University and in accordance with the criteria described in the NIH Guide for the Care and Use of Laboratory Animals. The IRB numbers are 201401112 and 201712020.

### Measurement of serum and urine parameters

Blood glucose levels were measured using an Accu-chek glucometer (Roche Diagnostics, Basel, Switzerland). The levels of blood urea nitrogen (BUN), serum creatinine (Scr) and micro-albumin were measured using the automated chemistry analyzer (Hitachi 917, Tokyo, Japan) and the commercial kits (Wako, Osaka, Japan).

### Enzyme-linked immunosorbent assay

To assess inflammation markers, serum samples were analyzed using mouse Magnetic Luminex Screening assays containing a premixed multi-analyte kit for murine TGF-β-1, TNF-α and IL-6 (R&D Systems, Minneapolis, MN, USA) on a Luminex 200 (Austin, TX, USA). The color generated was determined by measuring the optical density value at 450 nm with a spectrophotometric microtiter plate reader (Molecular Devices Corp., Sunnyvale, CA, USA).

### Cell culture

Normal rat kidney tubular epithelial cell line (NRK-52E) was obtained from the American Type Culture Collection (Rockville, MD, USA) and cultured in Dulbecco’s modified Eagle’s medium (DMEM) as previously described^32,43^. In the control group, the cells were treated with serum free DMEM medium only. In HG group, the cells were treated with 30 mM HG for 12, 24, 48, 72, and 96 h^32^.

### Transfection and RNA interference

We cloned hZnT8-EGFP and control EGFP vector, and human ZnT8 sequence was obtained from GenBank (NM_173851). hZnT8 was cut by restriction enzymes EcoR I and Xho I, after inserted hZnT8 into a pcDNA3.1/myc-hisA vector for sequencing. We transiently transfected NRK-52E cells with hZnT-8-pcDNA3.1/myc-hisA or pcDNA3.1/myc-hisAs and checked for the expression of hZnT8 at mRNA levels by RT-PCR. mZnT8 (NM_172816) siRNA (Stealth RNAi) was prepared by Invitrogen (USA). The sequence of the siRNA targets a specific sequence in mZnT8 mRNA, 5′-GAUCCAGUGUGCACAUUUATT-3′. The scrambled siRNA sequence used was 5′-UUCUCCGAACGUGUCACGUTT-3′. Cells were transfected with plasmid or siRNA using Lipofectamine 2000 (Life Technologies, USA) according to the manufacturer’s protocol. Western-blot analysis was performed to help optimize exogenous expression and RNAi silencing of ZnT8 with transfection.

### Assessment of cell viability

Cell viability was measured by quantitative colorimetric assay with 3-(4,5-dimethylthiazol-2-yl)-2,5-diphenyltetrazolium bromide (MTT) in 96-well plates. Briefly, at the indicated time after treatment, 10 μl MTT (final concentration, 500 μg/mL) was added to the medium and incubated at 37 °C for 3 h. The MTT solution was removed and 100 μl dimethyl sulfoxide (DMSO) was added to dissolve the colored formazan crystals for 15 min. The absorbance at 490 nm of each aliquot was measured using a Sunrise RC microplate reader (TECAN, Männedorf, Switzerland). Cell viability was expressed as the ratio of the signal obtained from treated cultures and control cultures.

### Immunofluorescence staining

Kidney tissues were anesthetized deeply with sodium pentobarbital and perfused with isotonic saline, followed by 4% paraformaldehyde in 0.1 M of phosphate buffered saline (PBS; pH 7.4). The kidney was removed and post-fixed with 4% paraformaldehyde overnight at 4 °C. Serial paraffin sections (4 μm) were prepared, dewaxed in xylene, and rehydrated using gradient alcohol solutions. The cryostat sections were then pre-incubated with normal donkey serum (1:20) for 1 h and incubated overnight at room temperature with anti-aquaporin-1 (polyclonal antibody, Abcam ab232399) at 1:100 and anti- E-cadherin (polyclonal antibody, Abcam ab1416) at 1:100; anti-Vimentin (polyclonal antibody, Abcam ab128507) at 1:100. After rinsing with PBS, the sections were incubated for 2 h with DAR-FITC (1:50) and Texas Red-DAM (1:50, Jackson ImmunoResearch) at RT. The sections were mounted and examined in a confocal laser scanning microscope (CLSM, SP2, Leica, Germany).

NRK-52E cells were fixed in 4% paraformaldehyde and per-mobilized in 0.1% Triton X-100, before they were respectively treated with primary mouse monoclonal anti-E-cadherin (E-ca) antibody (1:100, polyclonal antibody, Abcam ab1416), mouse monoclonal Vimentin antibody (1:100) and the sections were incubated for 2 h with DAR-FITC (1:50) and Texas Red-DAM (1:50) at RT. The fluorescent images were visualized with a Fluoview 300 fluorescence microscope (Olympus, Tokyo, Japan).

### Western blot analysis

Western blot analysis was performed as previously described^15,32^. Kidney tissues were homogenized in cold radio-immunoprecipitation assay lysis buffer, incubated on ice for 1 h, centrifuged at 12,000 × *g* for 20 min at 4 °C and then the supernatants were transferred to a clean tube. Protein concentrations were quantified using a Bio-Rad protein assay kit (Bio-Rad Laboratories, Inc., Hercules, CA, USA). Briefly, equal amounts of protein samples from kidney cortex fragments or NRK-52E cells were subjected to SDS-PAGE using 10% gradient Tris/glycine gels. Then, the proteins were transferred to polyvinylidene difluoride (PVDF) membranes (Millipore, Temecula, CA, USA). After blocking with 5% fat-free milk for 1 h, the blots were incubated with the following primary antibodies. The primary antibodies used in the present study were as follows: rabbit anti-ZnT8 (Mellitech, 1:200), Goat polyclonal anti-Vimentin (1:400, Abcam ab8978), mouse monoclonal anti-E-ca (1:400, Abcam ab1416), and mouse monoclonal anti-GAPDH (1:400; Santa Cruz Biotechnology, Inc. sc-47778), Smad2 (1:1000, Cell Signaling Technology, Danvers, MA, USA, 5339), Smad3 (1:800, Cell Signaling Technology, Danvers, MA, USA, 9523), p-Smad2 (1:1000, Cell Signaling Technology, Danvers, MA, USA, 18338), p-Smad3 (1:1000, Cell Signaling Technology, Danvers, MA, USA, 9520), TGF-β1 (1:400, Abcam ab92486). Following extensive washing in TBS-0.1% Tween 20, the membranes were then incubated with horseradish peroxidase-conjugated secondary antibodies, including rabbit anti-goat IgG (1:400) and rabbit anti-mouse IgG (1:400) overnight at 4 ˚C. Subsequently, the membranes were visualized using an enhanced chemiluminescence kit (Walterson Biotechnology Inc., Beijing, China) using the ChemiDoc™ XRS system with Quantity One software version 4.6 (Bio-Rad Laboratories, Inc.) and the G-BOX EF Chemi HR16 gel imaging system (Syngene, Frederick MD, USA). Following development, the band intensities were quantified using Image-Pro plus 6.0 analysis software (Media Cybernetics, Inc., Rockville, MD, USA). The blots were repeated at least three times for each condition.

### Zinc analysis

N-(6-methoxy-8-quinolyl)-p-toluenesulfonamide (TSQ) is a one-step staining procedure and by the stoichiometric formation of zinc. To analyze the distribution and concentration of zinc in the cells, TSQ fluorescence staining was analyzed as described previously^44^. Cultured NRK-52E cells were immersed in a solution of 4.5 μM TSQ (Molecular Probes, Eugene, USA) in 140 mM sodium barbital and 140 mM sodium acetate buffer (pH 10.5) for 1 min. TSQ binding was imaged with a fluorescence microscope.

### Statistical analysis

Statistical analysis was performed using SPSS (Version 18, IBM Corporation, Armonk, NY, USA). Data were expressed as means ± standard error (SEM). Variance was homogenous for use of standard ANOVA methodology. Individual comparisons were made using Tukey’s multiple comparison tests after statistical significance was established by ANOVA. *P* < 0.05 was considered as significance.

## Supplementary information

Supplemental Fig 1

Supplemental Fig 2

Supplemental Info
