# Supplementary figures and images for: Effects of ZnT8 on epithelial-to-mesenchymal transition and tubulointerstitial fibrosis in diabetic kidney disease
Source: Cell Death Dis. 2020 Jul 17;11(7):544. doi: 10.1038/s41419-020-2731-6 (PMC7367835; doi:10.1038/s41419-020-2731-6)

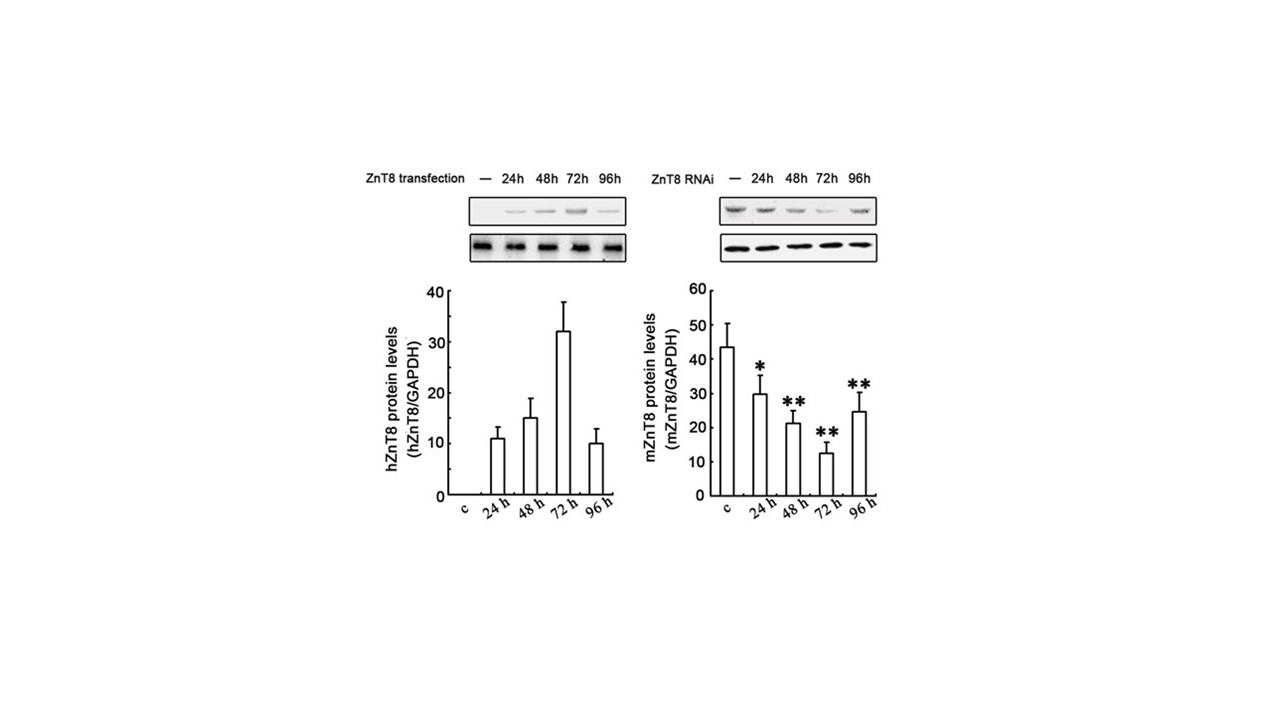

Supplement: Supplementary file 1 — Supplemental Fig 1 [file 41419_2020_2731_MOESM1_ESM.tif]

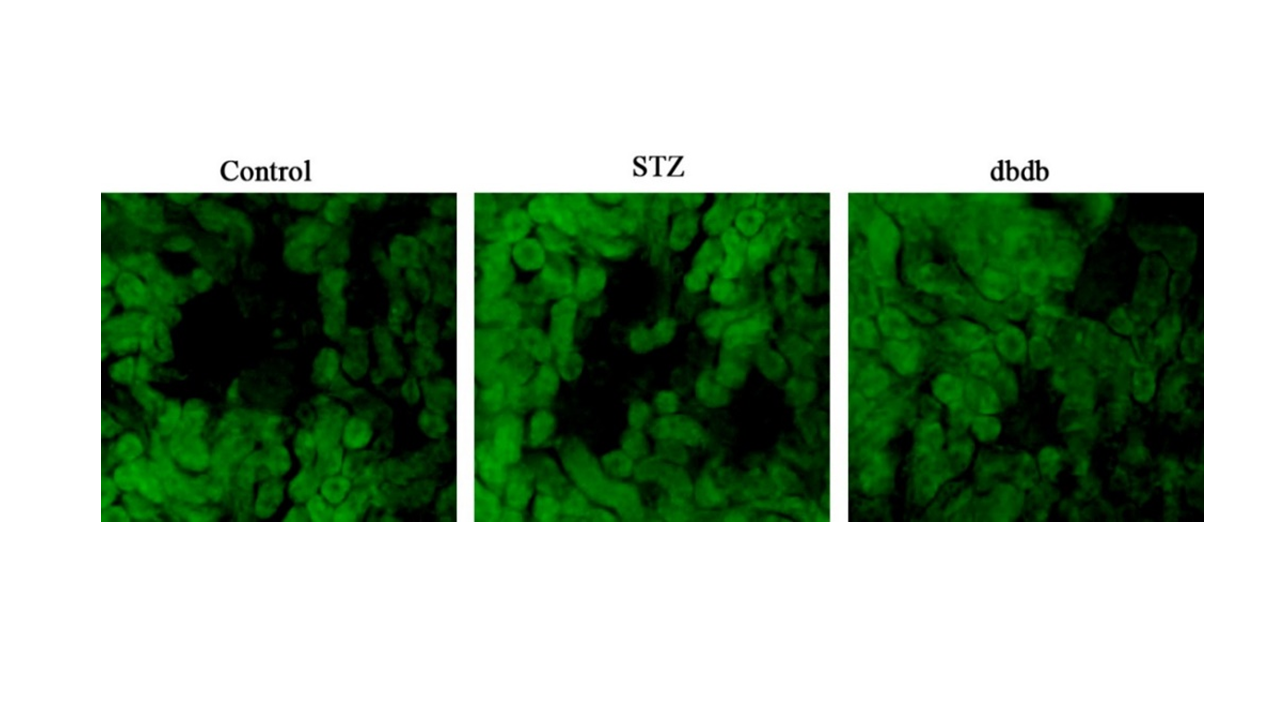

Supplement: Supplementary file 2 — Supplemental Fig 2 [file 41419_2020_2731_MOESM2_ESM.tif]
