# Supplementary material for: Effects of ZnT8 on epithelial-to-mesenchymal transition and tubulointerstitial fibrosis in diabetic kidney disease
Source: Cell Death Dis. 2020 Jul 17;11(7):544. doi: 10.1038/s41419-020-2731-6 (PMC7367835; doi:10.1038/s41419-020-2731-6)
Supplement: Supplementary file 3 — Supplemental Info [file 41419_2020_2731_MOESM3_ESM.docx]

**Supplemental Fig. 1**

**Western blotting analysis for ZnT8 transfection with hZnT8 expression vector and ZnT8 RNAi**

The strongest effects of ZnT8 in NRK-52E cells were observed after transfection for 72 h. Corresponding protein levels were assessed as the relative intensities using densitometry in Western blot (**P*<0.05 and ***P*<0.001 vs. Control group).

**Supplemental Fig. 2**

**Implication of ZnT8 expression in renal epithelial tubular cells in kidneys by immunofluorescence detection**

Immunofluorescence staining analysis implicated that ZnT8 was mainly expressed in renal epithelial tubular cells but very weakly in glomerulus or podocyte (Original magnification is x200).
